# Advances in small area population estimation in the absence of national census data

**DOI:** 10.1073/pnas.2413993123

**Published:** 2026-08-21

**Authors:** Andrew J. Tatem, Gianluca Boo, Heather R. Chamberlain, Christopher C. Nnanatu, Edith Darin, Douglas R. Leasure, Ortis Yankey, Assane Gadiaga, Sabrina Juran, Luis de la Rua Rodriguez, Jessica Espey, Attila N. Lázár

**Affiliations:** ^a^ WorldPop, Department of Geography and Environment, University of Southampton, Southampton SO22 5AT, United Kingdom; ^b^ Demographic Science Unit, Nuffield Department of Population Health, University of Oxford, Oxford OX1 1JD, United Kingdom; ^c^Latin America and the Caribbean Regional Office, United Nations Population Fund, Panama City 07144, Panama

**Keywords:** population modeling, census, geostatistical models, surveys, remote sensing

## Abstract

Population data at small area scales are essential for effective decision-making, influencing public health, disaster response, and resource allocation, among others. While national censuses remain the cornerstone of population data, they are often constrained by high costs, infrequent collection cycles, and coverage gaps, which can hinder timely data availability. To address these challenges, geospatial statistical approaches using limited microcensus surveys have been demonstrated as a reliable source, but the field has advanced substantially in recent years, with significant developments in both data sources and modeling methodologies. New approaches now leverage routine health intervention campaign data, satellite-derived settlement maps, and bespoke modeling approaches to produce reliable small area population estimates where enumeration is difficult or outdated. Various countries are applying these techniques to support census operations, health program planning, and humanitarian response. This manuscript reviews recent advances in “bottom-up” population mapping approaches, highlighting innovations in input data, modeling methods, and validation techniques. We examine ongoing challenges, including partial observation of buildings under forest canopy, population displacement, and institutional uptake. Finally, we discuss emerging opportunities to enhance these approaches through better integration with traditional data ecosystems, capacity strengthening, and coproduction with national institutions.

In a world where data increasingly shape policies, planning, and resource allocation, significant gaps persist in our knowledge of basic human demographics. Central to these gaps is the lack of small area data on population counts, arguably the cornerstone of planning and decision-making for governmental, nongovernmental, and private organizations (1–4). Vital registries (records of births, marriages, and deaths) and the population and housing census, hereafter referred to as “census,” are traditionally the most extensive demographic data sources. However, both face practical challenges that can limit their capacity to provide actionable data.

Censuses are typically conducted on a decadal basis, with results often released at a coarse spatial resolution, due to challenges with accurate geographic boundaries and to protect the privacy of individuals. Moreover, censuses require substantial human and financial resources, and ensuring full coverage can be particularly complex in remote or marginalized areas (5, 6). Data-rich countries may use population registers that are updated with administrative data collected at service points, but these require robust administrative systems which are limited or lacking in most low and lower-middle income countries (7). Urbanization, conflicts, migration, and climate change are leading to rapid changes in population distributions and demographics, with an increasing need for regularly updated demographic data. The United Nations’ 2030 Agenda for Sustainable Development (8) underscores the need for high-quality, timely, and small area population data. Half of all Sustainable Development Goal (SDG) indicators, such as those on poverty, health, education, and gender equality, require small area population data as a denominator, disaggregated by sex, age. However, millions of people remain invisible due to outdated censuses, undercounts, or inability to reach hard to count populations. Developing complementary population estimation methods becomes both a technical necessity and a human rights imperative to achieving the SDG principle of leaving no one behind.

Small area population estimation methods have increasingly been recognized as critical tools not only for addressing data gaps during intercensal periods but also for strengthening census operations themselves (9). These methods can help address coverage issues, cartographic updates, and support demographic reconciliation processes postenumeration. Importantly, such methods should not be seen as replacements for a national census, but rather as a complementary source that can enhance accuracy, equity, and responsiveness of statistical systems. The integration of these approaches into national data systems requires technical capacity, institutional coordination, and political will.

In 2018, Wardrop et al. (10) envisaged a census-independent “bottom-up” estimation approach to produce timely, subnational demographic estimates in countries where demographic data sources from censuses or registries were outdated, incomplete, or unavailable. The “bottom-up” approach leverages datasets from small area enumerations and high-resolution geospatial datasets derived from satellite imagery and other sources. The envisaged modeling methods aimed to estimate population counts and demographic characteristics at high spatial resolution across unsurveyed areas, together with measures of statistical uncertainty. Due to the variability in available demographic data, with differences in geolocation accuracy, sample sizes, and spatial coverage, bottom–up models were anticipated to require bespoke design and development.

Since the publication of Wardrop et al., conflicts, political changes, natural disasters, and the COVID-19 pandemic have resulted in census delays and cancellations in many countries, as well as increasing rates of undercounting in many censuses conducted in the 2020 round (11–13). Fueled by these issues and the substantial increase in geospatial and noncensus data availability, the bottom–up approach has seen important advances in the past 5 y. Here, we review the current state of these methods, with a focus on resource-poor settings. We present an overview of novel input demographic and geospatial datasets and modeling frameworks and the validation of outputs. Finally, we discuss current challenges and future opportunities facing the field.

## Approaches to Census-Independent Bottom–Up Population Estimation.

**Fig. 1.**
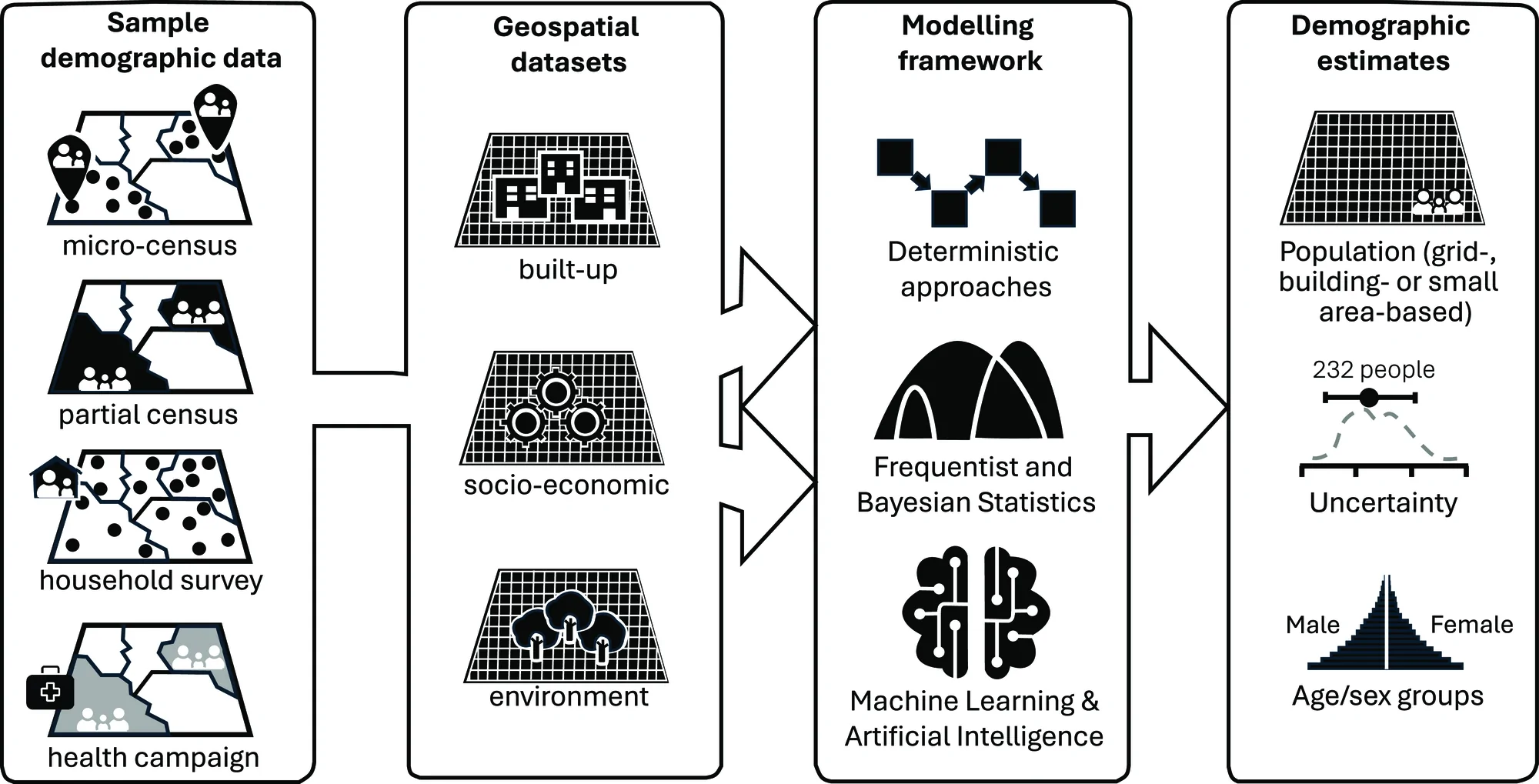
Components of the bottom–up approach to small area population estimation. Different sample demographic datasets are combined with ancillary geospatial datasets covering the entire study area in bespoke modelling frameworks to estimate population distributions and demographics.

This section reviews data sources and bottom–up modeling approaches for small area population estimation with examples. Fig. 1 provides an overview illustration of the main components and options for population modeling.

### Demographic datasets.

Bottom–up demographic estimation methods leverage observed population data to predict demographic attributes, such as population counts and age and sex structures, across unsampled locations. In recent years, digital collection and geolocation have become standard survey tools for censuses, household surveys, and health campaigns (11). This has resulted in significant improvements in attribute accuracy, enabling easier integration of these datasets into modeling processes. These demographic datasets can have different levels of spatial completeness (Fig. 1). Wardrop et al. introduced the concept of “microcensus” surveys for bottom–up population modeling, stressing the advantage of rapid data collection (10, 14–17). These surveys enumerate fully a relatively small, representative sample of well-defined small areas, typically of approximately three hectares, collecting geolocated total household size and age/sex distributions. They can accommodate more complex sampling designs, such as stratified and probabilistic samples (18), but do require considerable human and financial resources—albeit a fraction of a full census—and face similar challenges to a census in terms of inaccessibility and insecurity.

While some bottom–up model applications have relied on microcensus survey data, a wider set of models have since been developed to use recent, partial census enumeration to fill geographic gaps in census data collection (19, 20) or to support census planning processes (21). These data consist of demographic attributes associated with household coordinates or enumeration areas, have very large sample sizes, but do not provide a random sample of the population. Gaps in enumeration typically occur due to accessibility issues (6, 22), undercounts in certain types of communities, such as high-rise buildings and gated communities (23), and among certain demographic groups (5, 24). Population modeling approaches can help to identify and address such gaps.

Another demographic data source is household listings collected in preparation for national household surveys. These surveys are designed to be nationally representative by fully enumerating a stratified random sample at selected locations; in LMICs, they typically follow a stratified two-stage cluster sampling design. Repurposing the household listing offers the advantage of frequent and low-cost estimates updates (25–27). Such datasets typically include geographic data on the cluster locations and, in some cases, the coordinates of deidentified individual households and the sampling weights (28).

A different type of population data can be obtained from service planning and delivery activities targeting every household in a given area. For example, insecticide treated nets (ITNs) distribution campaigns often use a house-to-house distribution model, with relevant demographic attributes collected at the household level. Population counts collected as part of ITN distribution campaigns have been used in several bottom–up applications (29, 30), and have the advantage that semiregularly collected campaign data can enhance sample coverage and frequency, offering a practical complement to censuses or microcensus surveys. However, the household-level population data collected during distribution campaigns are not designed to be representative samples and details of the data collection protocol are often not documented. Thus, these datasets are more prone to specific biases, such as omitted or duplicated households, or inflated household sizes, requiring careful quality assessment and adjustment.

### Geospatial datasets.

Geospatial datasets typically representing phenomena related to population distribution are an essential component of bottom–up population modeling. Geospatial datasets have advanced substantially since Wardrop et al. in quality, spatial detail, and update frequency, particularly those derived from satellite imagery. These developments are associated with increasing computing power and advances in machine learning algorithms, enabling classification and feature extraction at scale. To be able to predict into areas missing demographic data, geospatial datasets must have complete coverage of the area of interest. Such ancillary datasets are summarized within small areas, administrative or census units, or in a regular gridded format, with spatial resolution matching the target population estimates—e.g., approximately 10 m (31) or 100 m (32).

The most important geospatial datasets for population modeling describe built-up areas (33) that have seen major advances in recent years: now mapping individual buildings (e.g., footprint outline and estimated height) or settled areas with near-global coverage from multiple sources (31, 34–37). Building footprints can be used to derive, for example, building count, area, perimeter, volume, and other morphological characteristics that can be valuable predictors (e.g., settlement types, neighborhood types, residential/nonresidential classes) (31, 38–42). Building footprints are now becoming available with annual timestamps, such as the Google 2.5D Open Buildings dataset, enabling temporal alignment with demographic datasets (43).

Other geospatial datasets leverage the growing availability of volunteered geographic information (VGI). OpenStreetMap for example (44), provides detailed geographic locations for different point of interests (e.g., health and education facilities) associated with population distribution (45). Regional and global datasets mapping health and education facility (46), violent events (47), and building damage (48, 49) also offer potential value as predictors of population at small area scales. These point location datasets can be summarized using focal windows to map push-pull effects of different phenomena on population distributions. Additionally, digital trace data from mobile devices have been used as indicators of population presence, including call detail records (50) and smartphone app data (51).

Population distributions are also associated with environmental factors, such as topography, climate, water bodies, and land cover (33)—available globally at high spatial resolution and with frequent updates (52). Similarly, remote sensing images of nighttime light brightness can capture features relating to human presence, such as areas with electrification and fires, though need careful processing to remove features like gas flares and volcanic eruptions (53).

### Modeling frameworks.

Wardrop et al. suggested simple regression and geostatistical approaches as potential bottom–up modeling frameworks. Subsequent approaches to bottom–up population estimation have been developed to accommodate available input data, including deterministic approaches, frequentist and Bayesian statistical models (including further geostatistical developments), machine learning, and AI. These approaches all leverage relationships between the demographic and geospatial datasets to estimate demographic attributes at both sampled and unsampled locations.

The simplest bottom–up model combines building or settlement information with average values of population densities (e.g., people per km^2^ or people per building) applied uniformly across the study area. Such models can be improved when the calculation differentiates residential/nonresidential buildings, households per building, or the average household size (54). While accounting for local variations might be limited in such approaches, the availability of high-resolution observed building density, building height, and building type datasets can dramatically improve the precision of outputs compared to solely using the mapped presence of built settlement (42).

Leasure et al. (16) used Bayesian hierarchical models to estimate population counts as a Poisson process using the product of settled area and estimated population densities. Population densities are modeled as a lognormal process to better capture variability in the input data. The model is designed to draw information from different scales, such as coarse administrative units (e.g., regions or districts) or functional areas (e.g., settlement classes), to define the model parameters, including intercepts, covariate (i.e., geospatial data) effects, and variance terms (16, 26). Hierarchical intercepts account for stratified sampling designs as well as potential patterns in population density. Moreover, by using settlement type or administrative unit fixed effects, key information is shared among various spatial scales, thereby, leveraging observations from neighboring clusters to predict population densities across clusters with no observations. Finally, the settlement-class-specific variance term captures the residual variation. Prior distributions in these models are informed by established empirical regularities—for example, the approximately log-normal distribution of population density across settlement types and the expected ordering of densities by urbanization class—with sensitivity analyses used to assess robustness to prior specification. More recent hierarchical model designs now use building footprints to define these parameters enabling more accurate population distribution predictions (17, 19, 25), with further developments allowing joint estimation of building count and population distributions (18, 55).

To enable the use of household surveys for population modeling (25) required a crucial study by Leasure et al. (28) that demonstrated that a population-weighted sample (i.e., national household surveys with a probability proportional to size design) results in biased estimates of population densities. They explored various Bayesian approaches and concluded that weighted-likelihood models can recover unbiased estimates of population densities and population totals from a nonrandom, population-weighted sample.

Spatial variation in bottom–up population models is needed, because populations are not randomly or uniformly distributed in space, and thus ignoring spatial autocorrelation can lead to biased estimates. Nnanatu et al. (27) used Bayesian geostatistical models to explicitly account for spatial autocorrelation not captured by covariates through leveraging distance-dependent covariance matrices. This included a two-stage Poisson-Gamma geostatistical regression (itself a hierarchical model). Similarly to the hierarchical models, this design also assumes that population density is related to a set of geospatial covariates through a linear regression model of covariate effects, but estimates population counts as the product of the predicted population density and the corresponding building counts. However, the Poisson-Gamma approach utilizes a Gamma probability density instead of the lognormal of the hierarchical models above to model the population density. Geostatistical models specify spatial autocorrelation using a triangulation (or mesh) of the entire continuous spatial domain. These models have been also applied where, due to permanent canopy covers, building footprints were only partially available (29).

Machine learning (ML) and AI can be used to estimate intercensal population counts at high spatial resolution directly from satellite imagery. Landscan HD (56) uses a data fusion approach to merge relevant land use and building layers in a deep learning framework to generate gridded population estimates. Convolutional neural networks with visual geometry group architecture and other ML models (e.g., gradient boosting) are also used for deep learning directly from a concatenation of low-resolution Landsat-7/8, Sentinel-1, and satellite-derived night-time light intensity (57–60). These new methods leverage frequent and freely accessible satellite imagery updates to produce frequently updated population estimates. The use of very high-resolution satellite imagery, such as the Maxar VIVID 2.0, in conjunction with a small sample of ground truth dataset (i.e., microcensus), appears promising to help with the identification of inhabited areas and nonresidential buildings, and thus estimating population distributions more accurately (61).

Most of the bottom–up modeling frameworks outlined above estimate the total population distributions, but service planning, resource allocation, and exposure assessments typically require data on specific sex and age subgroups. The most widespread method for age- and sex-disaggregation uses observed or projected subnational population pyramids to deterministically disaggregate total population count estimates (62). However, when the input demographic datasets contain age and sex information, bottom–up models can also approximate sex and age subgroups. There are examples where the proportion of the population under the age of 5 y is estimated at high spatial resolution using a Bayesian spatiotemporal model (63), or where population pyramids are estimated as Dirichlet-multinomial processes (17), or by using a series of Bayesian binomial processes (64). Field campaigns and development planning often require household level information that can also be modeled at small area scales using Bayesian geostatistical and hierarchical models (27, 65) or household characteristics modeled through machine learning methods (66).

## Outputs and Validation.

**Fig. 2.**
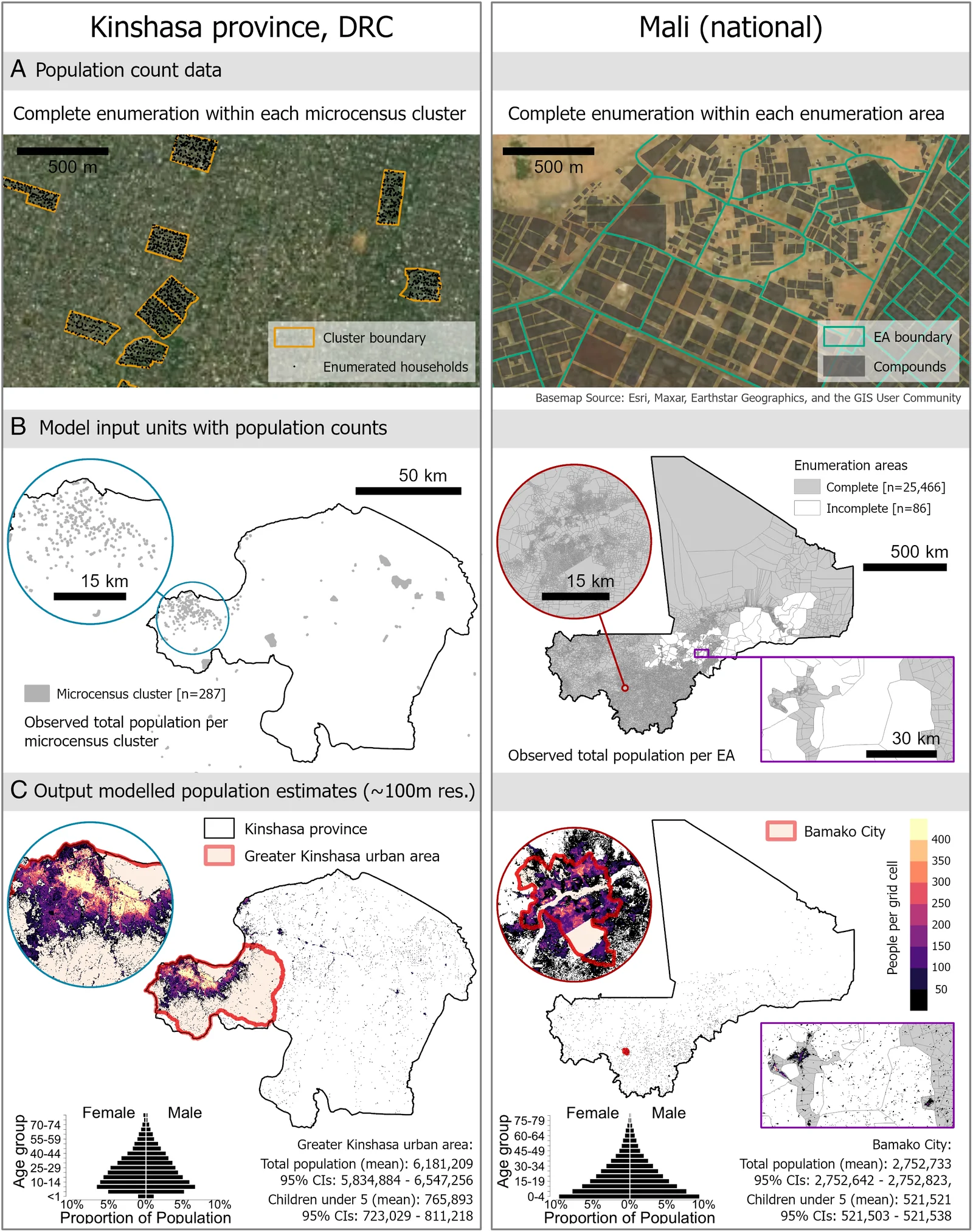
Example bottom–up model applications: field observations (panel *A*), input data (panel *B*), and gridded population estimates with age/sex and uncertainty information (panel *C*). Data sources: Democratic Republic of the Congo (DRC) microcensus (75), DRC population estimates (17), Mali census cartography (76), Mali population estimates (21).

Bottom–up population estimates have been typically produced for regular grid cells, with a spatial resolution of approximately 100 m (Fig. 2). This format provides a continuous surface enabling the flexible aggregation to different geographic decision-making units, such as administrative districts, health zones, or summarized by features of interest, such as urban extents, flooded areas, or hurricane tracks. This format also facilitates integration with other geospatial datasets, for instance, health facilities, conflicts, schools, and polling booths, for geographic accessibility analyses. Furthermore, bottom–up population estimates have been used to calculate social-distancing indicators (67), map out-of-school children (68, 69), support disaster response (70, 71), create survey sampling frames (72, 73), and plan vaccination campaigns (74).

An understanding of the confidence in modeled population estimates is important for decision makers and planners. ML models generally capture model uncertainty by using bootstrap resampling of the training data, that is to repeatedly draw random samples from a single original training dataset to create many new, simulated datasets (59, 66). This tests that the model can generalize well to unseen areas. It should be noted, however, that standard bootstrap resampling may not provide valid uncertainty estimates for all ML algorithms, particularly those with complex regularization structures; ensemble methods or other resampling strategies may be more appropriate in such cases. Other uncertainty estimation uses grid search, a hyperparameter optimization technique that exhaustively tries every possible combination of hyperparameter values from a user defined grid (61). Validation of ML models include leave-one-out cross-validation, quantifying how close the observed and predicted probabilities or counts are to each other.

Bayesian methods quantify statistical uncertainty. Model assumptions, guided by expert knowledge and data availability can be tested in simulation studies (28, 29), assessing input data and model structure. Model uncertainty is quantified by assessing the spread of the parameters’ posterior distribution. These posterior distributions are used to calculate metrics such as the mean value of the predicted population counts, the SD, and 95% credible intervals (CIs). Model uncertainties are propagated through the Bayesian CIs. For example, Boo et al. (18) propagated uncertainties of observed buildings through the modeled population density and the gridded population estimates to the various levels of spatial aggregations of the outputs. The results highlighted that the uncertainty in the modeled estimates varies spatially and increases with granularity. Knowledge of spatial variation of uncertainties can enable more informed decisions. For example, where there is a specific target for vaccination coverage of children under 5 y e.g., 90%, the upper CI can provide an estimate of the maximum number of children expected to be found, guiding vaccine amounts required to achieve target coverage. Spatial maps of uncertainty can also be used to prioritize where additional demographic data collection can maximize improvement of modeled estimates.

Model validation of population models is generally done through model diagnostics, goodness-of-fit assessment, and external comparisons against an independent set of observations. Model diagnostics include among others: checking assumption of linear regression, checking parameters convergence, posterior predictions, grid-cell predictions, undertaking cross-validation to check the robustness of the design, as well as uncertainty visualization (16). Goodness-of-fit assessments evaluate differences between the population estimates and the input data through different metrics, such as RMS error (RMSE), mean average error (MAE), correlation coefficients (Person’s correlation or R^2^), and spatial autocorrelation (global and local Moran’s I) (27). Standard metrics such as RMSE and R^2^ do not account for spatial autocorrelation, which is ubiquitous in population data; spatially aware metrics and cross-validation designs that respect spatial structure are therefore preferable where feasible (27).

Plausibility checks against population projections or other external sources of population data are also widely used to examine estimates of population models. In the first national-scale validation of census-independent bottom–up estimates against a subsequent census, Chamberlain et al. (77), for example, compared the 2022 national Zambian census counts with bottom–up modeled estimates representing the year 2019. Their analysis demonstrated strong correlation (R > 0.95 at ward and district level), with a tendency to exceed census counts that was substantially reduced when restricting comparisons to residential census household locations—highlighting the importance of improved built-settlement stratification in model inputs. This study represents a critical strengthening of the evidence base for bottom–up modeling, while also identifying clear priorities for methodological development. To counter external data triangulation challenges more generally, Darin et al. (55) sampled the 2018 Colombian census data to create an artificial dataset and thus making the sample and the validation data directly comparable. They tested two Bayesian and one ML model for accuracy and found that the ML model was the best suited for unbiased, complete observations, whereas the Bayesian methods were the best for coarse or biased observations and for sparsely populated regions.

## Challenges and Future Directions.

**Fig. 3.**
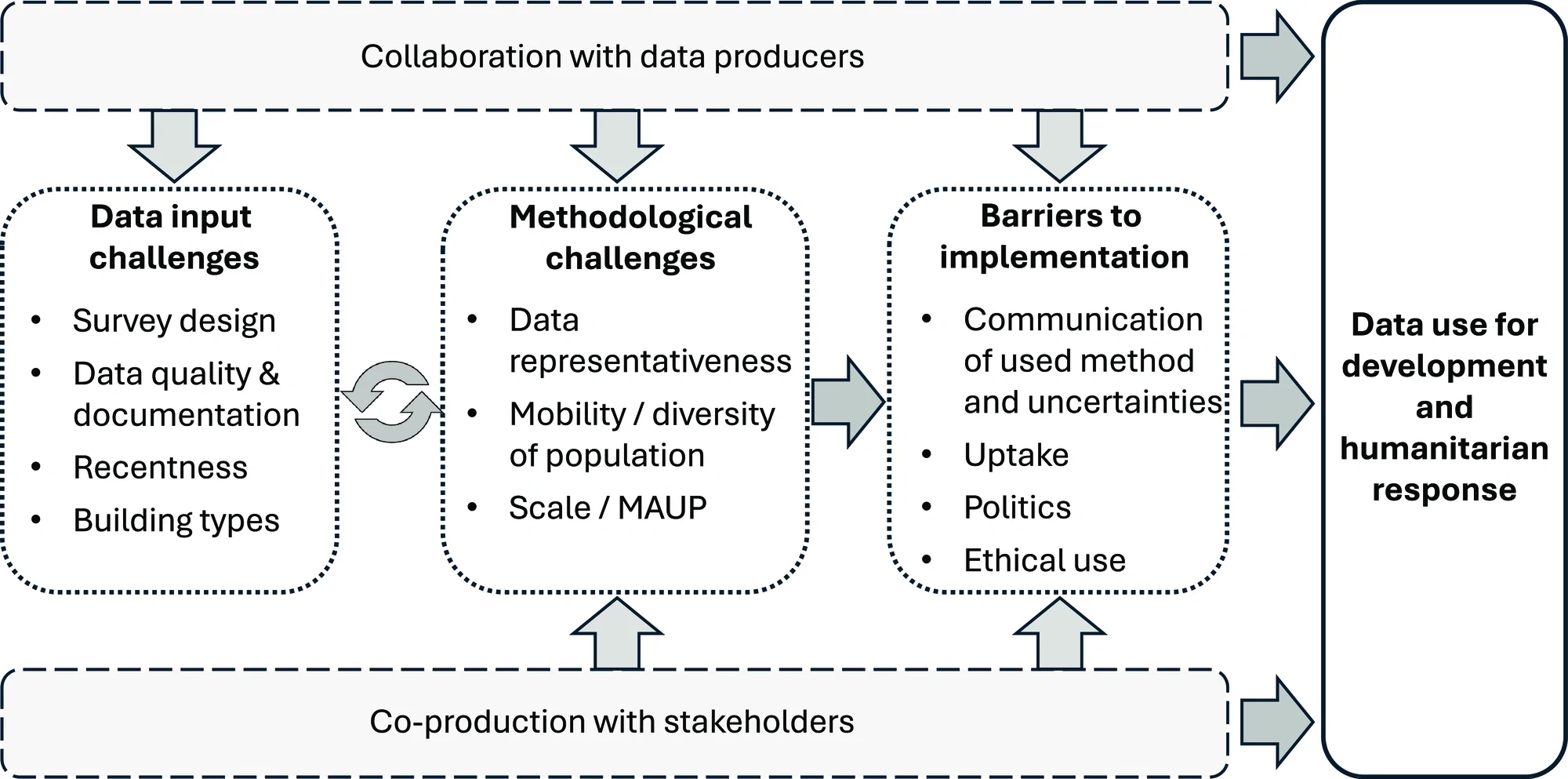
Challenges (white boxes) and opportunities (gray boxes) toward sustainable data production and use. MAUP = Modifiable Areal Unit Problem.

Recent advances in data sources and methods have addressed many challenges outlined by Wardrop et al. (10), but some remain, and new ones have emerged as applications involve increasingly diverse data sources. These challenges can be summarized into i) data input challenges, ii) methodological considerations, and iii) implementation barriers (Fig. 3).

## Data Input Challenges.

Challenges persist regarding demographic data quality and suitability. Field data collection has benefited from GPS-enabled devices that facilitate navigation and location recording, but practical limitations remain, especially around accessibility and safety concerns for enumerators (78, 79). Local community skepticism or distrust around data collection efforts can result in nonresponse or incomplete coverage, particularly among hard to count populations (80). Enumeration data collected through health distribution campaigns can result in biases in numbers upward or downward, depending on incentives in receiving services or resources (81). Though field data collection software enables near real-time quality checks, data quality ultimately depends on survey design, field protocols, data management, and team training (79). This underscores the importance of thorough documentation of survey methodologies, particularly as financial resources for field data collection become increasingly constrained (82).

For increasing the temporal relevancy of population estimates, digital trace data from mobile phone call detail records (83), smartphone location history (84), mobile applications (85), and social media platforms (86, 87) have demonstrated potential for measuring population distributions and movements in near real-time. These data sources are valuable for their volume and frequency of updates, though spatial resolution varies along urban–rural gradients and representativeness across population subgroups remains uncertain. Methods to correct for these biases using survey data are emerging (88, 89), but access challenges due to private ownership persist. Crucially, the representativeness biases in digital trace data are not random. Underrepresentation is structurally correlated with lower income, female gender, older age, and rural location, which typically represent the demographic groups most likely to be missed in conventional censuses. This means digital trace data cannot easily fill census gaps without active bias correction, and the compounding of these biases with temporal data gaps deserves closer attention. The areas hardest to enumerate are also those for which data age most rapidly and where intercensal population change is greatest, making reliable estimation most difficult precisely where it is most needed.

Despite major developments in building footprint and settlement datasets, in terms of global coverage and availability, challenges remain around temporal and spatial coverage due to cloud cover, tree canopy obstruction, and certain areas not being systematically mapped in open datasets due to conflicts or other sensitivities. Documentation of these datasets can be limited, making it difficult to ascertain the currency of imagery used in their construction, classification criteria, and methodological assumptions. Substantial variations exist between datasets (90–92), likely translating into significant differences in bottom–up population estimates.

## Methodological Considerations.

Estimating remote rural population numbers at small area scales remains challenging (93). Moreover, at the other end of the population density spectrum, the accurate estimation of informal settlement population numbers in urban areas presents particular difficulties (94). Combining satellite imagery data, statistical modeling, and community engagement is however offering a promising solution (95). Mobile populations also present a significant challenge. According to UNHCR, 117.3 million people worldwide were forcibly displaced at the end of 2023, excluding those displaced by environmental disasters and climate stressors (96). These rapid-onset events driving displacements and other localized movements, including nomadic populations, are generally not captured in the types of static population models primarily discussed in this paper. Recent model applications have attempted to address this by creating displacement-adjusted layers that redistribute populations based on known displacement patterns (97). Feature extraction algorithms have been developed to map structures in refugee camps (98), and humanitarian administrative data have been integrated with satellite-derived building footprints to map registered refugee populations (99). However, many types of mobile populations remain inadequately represented in both current bottom–up modeling approaches and traditional demographic datasets in general.

Spatial autocorrelation in input data can require attention as it can alter observation variance and increase sensitivity to certain covariates (100, 101). Furthermore, spatial autocorrelation is particularly important when there is spatial dependence in the data that cannot be accounted for by the model covariates, i.e., when spatial autocorrelation remains in the residuals of a fully specified model. The use of geostatistical models that handle spatial autocorrelation can address this.

The modifiable areal unit problem (MAUP) is the statistical bias arising from the choice of spatial units used to summarize point-based observations (102). MAUP represents a crucial modeling consideration, particularly when the spatial scales of observations differ from modeling units (e.g., grid cells). This mismatch, often called ecological fallacy, can reduce explained variance, increase residual spatial autocorrelation, and potentially lead to erroneous conclusions (103, 104). Simulation studies can help determine appropriate scale choices (105), while Bayesian models that incorporate the sampling frame components can better account for such population distribution uncertainties (103). It is important to emphasize that bottom–up models are specifically designed to avoid this problem. Rather than estimating parameters from administrative-level aggregations and then disaggregating to grid cells—the step that would incur ecological fallacy—bottom–up approaches typically specify population density as a function of cell-specific covariates, most importantly building footprints, and estimate at or near the resolution of those covariates. Administrative-level training data are used only where necessary to match available demographic observations, with parameters explicitly defined at the cell level. Geostatistical formulations additionally model residual spatial structure not captured by covariates, further grounding predictions at the scale of the observations themselves (27, 29). This structural distinction from top–down disaggregation approaches is fundamental to the census-independence and spatial validity of bottom–up methods.

## Implementation Barriers and Opportunities.

While a growing number of applications show that bottom–up population estimates provide valuable support for planning and decision-making during intercensal periods, they often face barriers to acceptance by decision makers, who may be reluctant to use modeled data and to collaborate with external partners to produce population estimates that may conflict with official projections. Population statistics have significant implications for resource allocation (106), political representation (107), and national economic performance and SDG indicators (108), making their official endorsement politically sensitive but key to ensure data ownership (109). To facilitate greater governmental uptake, several approaches have shown promise. There is a need to have a supportive data policy and regulatory environment, which is often a stumbling block for national statistical offices and ministries to collaborate with external third parties. Furthermore, enhanced communication of model methods, results, and associated uncertainties (110) and crucially, codevelopment with decision-makers and local ownership of the modeling process (111). Local ownership ideally means that when external partners are brought in to support the design and use of these new methods, the approach is codeveloped with end users, such as national statistical offices, and is accompanied by appropriate capacity strengthening for sustainable data generation and use (112). Medium- to long-term investment is needed to develop and retain institutional skills and strengthen national university programs to cultivate the next generation of geospatial statisticians. Moreover, building links with the rapidly growing ML and AI expertise in start-ups and NGOs can drive local capacity strengthening and sustainable adoption.

A growing concern is that high-resolution population estimates might expose vulnerable groups to unwanted surveillance or security risks and might impact equitable resource allocations (113, 114). Ensuring responsible use of digital technologies, as well as the publication and use of population data are crucial. Good practice includes seeking approval from independent ethics boards of the data provider, government and/or universities, deidentification of observations, and publishing of only aggregated statistical estimates. At the UN level, the publication of guidance for countries utilizing these modeling approaches and population estimates on effective practice and good governance should be considered. It would empower countries to embrace alternative methodologies to fill crucial population gaps, while showing how such modeling approaches can be appropriately and confidently applied.

## Conclusions

Reliable, timely, and detailed population estimates are increasingly required for effective decision-making, development planning, SDG reporting, operational campaign planning, and humanitarian response. Since Wardrop et al. (10) presented their perspectives on census-independent population estimates, significant advances have been made in both data sources and methodological approaches. The emergence of building footprint datasets, complex data integration with Bayesian and ML modeling frameworks, and better quantification and communication of statistical uncertainty have enabled improved bottom–up population estimates and uptake. The methodological and data infrastructure for census-independent population estimation has now matured sufficiently that the primary barriers to wider impact are no longer predominantly technical, but institutional: the availability of in-country capacity, supportive data policy environments, and coproduction frameworks that embed these methods sustainably within national statistical systems. Continued methodological development should be pursued alongside the challenging work of making existing methods operational, trusted, and sustainable. For practitioners, the choice of modeling framework depends principally on data availability and quality. Deterministic building-density approaches are appropriate where only aggregate building counts are available; Bayesian hierarchical models are preferable where sample observations are sparse, spatially biased, or require uncertainty propagation; and machine learning approaches are best suited where large, spatially complete training datasets exist. Open-source software implementations are available for several of these approaches, including peanutButter (54), the WOPR API (115), and jollofR (64).

In Burkina Faso (116), Mali (117), Papua New Guinea (118), and South Sudan (119), bottom–up population estimates have been adopted by national statistical offices for national planning. In Colombia, the national statistical office implemented these methods to address census enumeration gaps (20). UN agencies including UNFPA and the United Nations Children’s Fund (UNICEF) are actively promoting and applying these approaches (9, 120), as well as practical implementation tutorials (121, 122) to support field operations and data sustainability by building in-country capacity to use and update the models.

Population models successfully capture population distributions when using good quality, representative observations. Challenges, however, remain in capturing urban diversity, informal settlements, mobile populations (e.g., homeless, nomadic), and areas with persistent heavy vegetation. Looking forward, the field is poised to benefit from increasing availability of large-scale nontraditional demographic (e.g., digital trace) data, advances in statistical methods, including ML and AI to leverage such big data sources rapidly. Caution is always needed however to address biases in survey data, digital traces, and other inputs to models.

As demographic data gaps increase (123), the complementary role of bottom–up population estimation approaches will likely expand. Their ability to address some of the technical challenges likely to be faced in the 2030 census round (11), and their integration with national demographic data systems offers a path toward more responsive and cost-efficient statistics, particularly in contexts of census disruption, undercoverage, or demographic reconciliation for planning and monitoring.

Continuing methodological advances and stronger partnerships and coproduction between data producers, modeling teams, and stakeholders are needed to further enhance the utility and acceptance of these approaches. For example, in Latin America and the Caribbean, a collaborative partnership model involving UNFPA, Economic Commission for Latin America and the Caribbean (ECLAC), regional statistical entities such CARICOM, and national statistical offices has proven effective (124). These partnerships leverage contextual knowledge and statistical expertise to codevelop robust estimates. Such rigorous partnerships enable reliable applications, proper institutionalization of the methods, and the achievement of data-driven, inclusive development.

## Data Availability

There are no data underlying this work.
